# Review: Pelvic nerves – from anatomy and physiology to clinical applications

**DOI:** 10.1515/tnsci-2020-0184

**Published:** 2021-10-08

**Authors:** Ibrahim Alkatout, Thilo Wedel, Julian Pape, Marc Possover, Juhi Dhanawat

**Affiliations:** Department of Gynecology and Obstetrics, University Hospitals Schleswig-Holstein, Campus Kiel, Arnold-Heller Str. 3, Building 24, 24105 Kiel, Germany; Department of Anatomy, Institute of Anatomy, Center of Clinical Anatomy, University Hospitals Schleswig-Holstein, Campus Kiel, Otto-Hahn-Platz 8, 24118 Kiel, Germany; Possover International Medical Center, Zürich, Switzerland; Department of Gynecology, University of Aarhus, Aarhus, Denmark

**Keywords:** pelvis, pelvic neuroanatomy, pelvic neurophysiology, chronic pelvic pain, hypogastric nerves

## Abstract

A prerequisite for nerve-sparing pelvic surgery is a thorough understanding of the topographic anatomy of the fine and intricate pelvic nerve networks, and their connections to the central nervous system. Insights into the functions of pelvic nerves will help to interpret disease symptoms correctly and improve treatment. In this article, we review the anatomy and physiology of autonomic pelvic nerves, including their topography and putative functions. The aim is to achieve a better understanding of the mechanisms of pelvic pain and functional disorders, as well as improve their diagnosis and treatment. The information will also serve as a basis for counseling patients with chronic illnesses. A profound understanding of pelvic neuroanatomy will permit complex surgery in the pelvis without relevant nerve injury.

## 1 Introduction

### 1.1 Overview

“In surgery, anatomy is able to illuminate exactly that which remains otherwise concealed to the clinician and yet constitutes the essence of our courage as surgeons, namely our knowledge of what lies below and not merely a vague suspicion of what could lie below” (Ibrahim Alkatout, personal communication).

**Figure 1:**
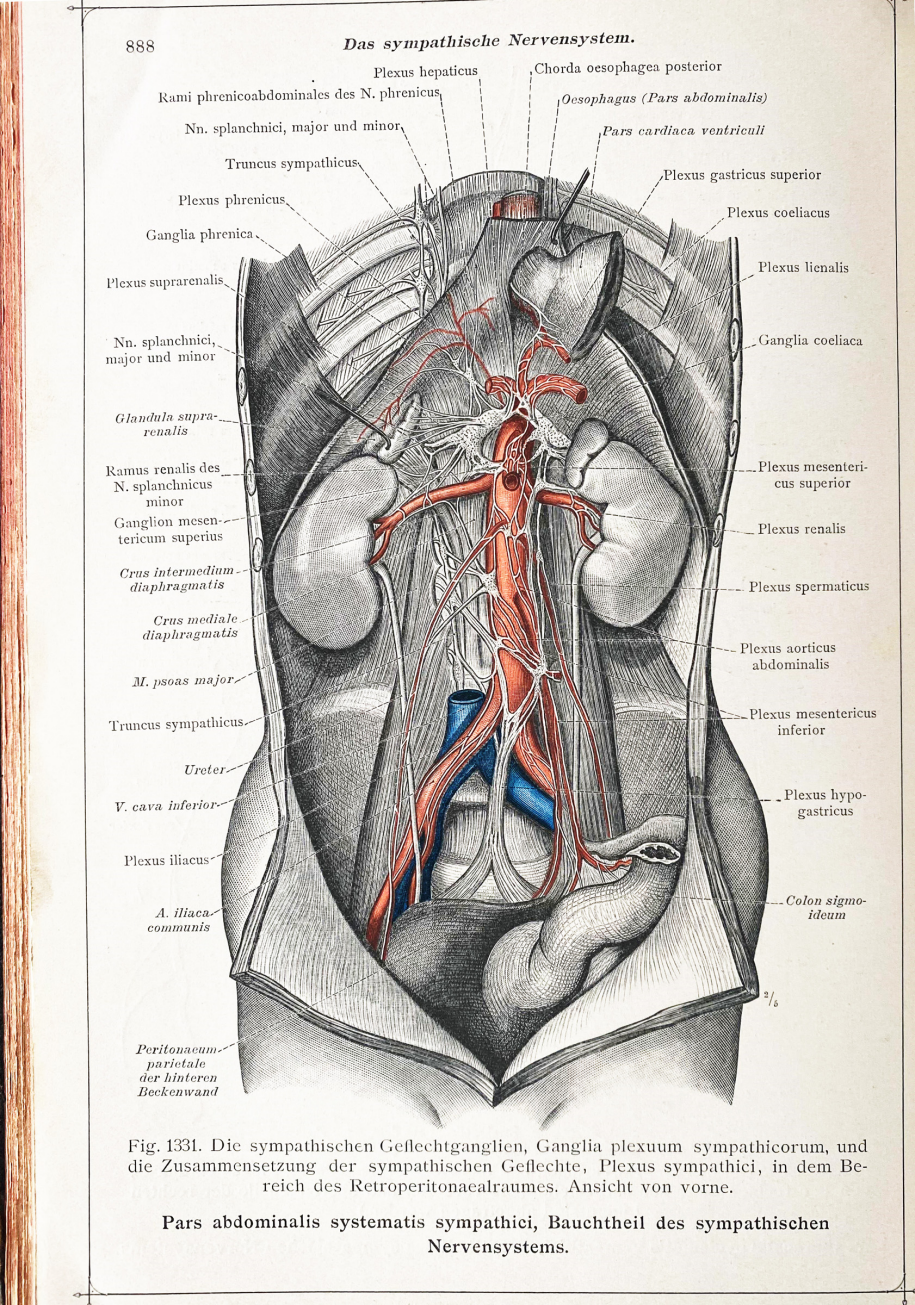
Anatomical illustration of the abdominal sympathetic system in an ancient anatomical atlas (Dr Carl Toldt, Berlin and Vienna, 1903).

John Newport Langley coined the term *autonomic nervous system* in the late nineteenth century and discovered – besides the enteric nervous system – the two major components of the autonomic nervous system, namely the so-called sympathetic system and the cranial and sacral parts of the parasympathetic system [1,2]. Since its first description, this classification has not changed much in terms of physiology and anatomy. The gross morphology of autonomic nerves has been depicted in several anatomical illustrations over the last centuries (Figure 1). However, the functional importance of pelvic autonomic nerves was realized due to complications like genitourinary and anorectal dysfunctions after pelvic surgeries. Thus, the concept of nerve-sparing surgeries came to prevent these nerves from getting significantly damaged not only in oncology but also in benign radical pelvic surgery.

### 1.2 Surgical aspects (nerve-sparing surgery)

Okabayashi radical hysterectomy procedure was reported in 1921 [3]. Due to functional complications, the need to modify the procedure to spare pelvic nerves had been realized [4]. Thereby, the procedure has been modified several times by surgeons over the years and the well-described nerve-sparing radical hysterectomy was first published by Fujii et al. [5,6,7,8]. In 1982, Patrick Walsh and Pieter Donker proposed a nerve-sparing radical prostatectomy for prostate cancer as a means of preventing sexual dysfunction. A detailed anatomical description of pelvic autonomic nerves in men was provided by Donker in 1986 [9,10]. Total mesorectal excision (TME) was introduced for the treatment of rectal cancer and promoted by Richard Heald; the cancer-bearing rectal tissue and the surrounding mesorectal package are removed along the predefined embryological planes, leaving the pelvic autonomic nerves intact [11].

Nerve-sparing surgery in the pelvis is used not only for oncological purposes but also for the treatment of benign diseases in the pelvis. Deep infiltrating endometriosis frequently calls for deep dissection of pararectal and paravaginal spaces in order to remove nodules lying adjacent to autonomic nerves [12]. Pelvic autonomic nerves may also be damaged during urogynecological procedures, such as sacrospinous ligament fixation, sacrocolpopexy, or rectopexy.

Symptoms of intraoperative nerve injury may be observed immediately after surgery or may be manifested several years later [13]. To avoid injury and subsequent morbidity, pelvic surgeons must be thoroughly familiar with the topographic anatomy of pelvic nerves. Many recent advancements have been made to understand pelvic neuroanatomies like computer-assisted dissections and 3D reconstruction of anatomy [14,15,16,17]. Despite this, exact identification and safe preservation of pelvic autonomic nerves still pose a challenge for pelvic surgeons.

### 1.3 Functional aspects

Along with pelvic neuroanatomy, neuropathophysiology is also important, especially in the context of visceral pain. The pathophysiology of pelvic visceral pain is complex and has not been completely understood. Both central and peripheral nervous systems play a role in chronic pelvic pain (CPP). The somatic and autonomic nervous systems of the pelvis are involved in the mediation of symptoms. Multiple mechanisms like viscerosomatic and viscerovisceral convergence, peripheral and central sensitization, referred pain, growth of new nerve fibers and receptors, imbalance of neurotransmitters, chronic inflammation, autoimmunity, expansion of somatosensory cortex, pain memory have been proposed to explain this complex field of medicine, which has been discussed below [18,19,20,21,22,23].

Immunohistochemical studies have added a major advantage to our understanding of the physiology of these nerves and their cross-connections [14,15,16].

Neuropelveology, now a well-developed branch of neurological sciences, deals with pelvic nerves, the generation of pain, and putative sites of lesions along the nerve pathway. Knowledge of these aspects enables the clinician to provide specific treatment [24]. The diagnosis is confirmed by clinical investigation and imaging modalities. Appendix A summarizes the history and examination to be done to reach a neurological diagnosis [24,25].

The treatment of CPP in the last decades has included sympathectomy, nerve blocks, neurolysis, presacral neurectomy, botox injections, and laparoscopic uterine nerve ablation (LUNA), which has not proven to be effective [26] and, more recently, pelvic neuromodulation [27,28,29].

In this article, detailed anatomy and physiology of pelvic nerves have been discussed with the aim for better surgical practice and understanding of the complex pathophysiology of pelvic diseases.

## 2 Neuroanatomy of the pelvis

**Figure 2:**
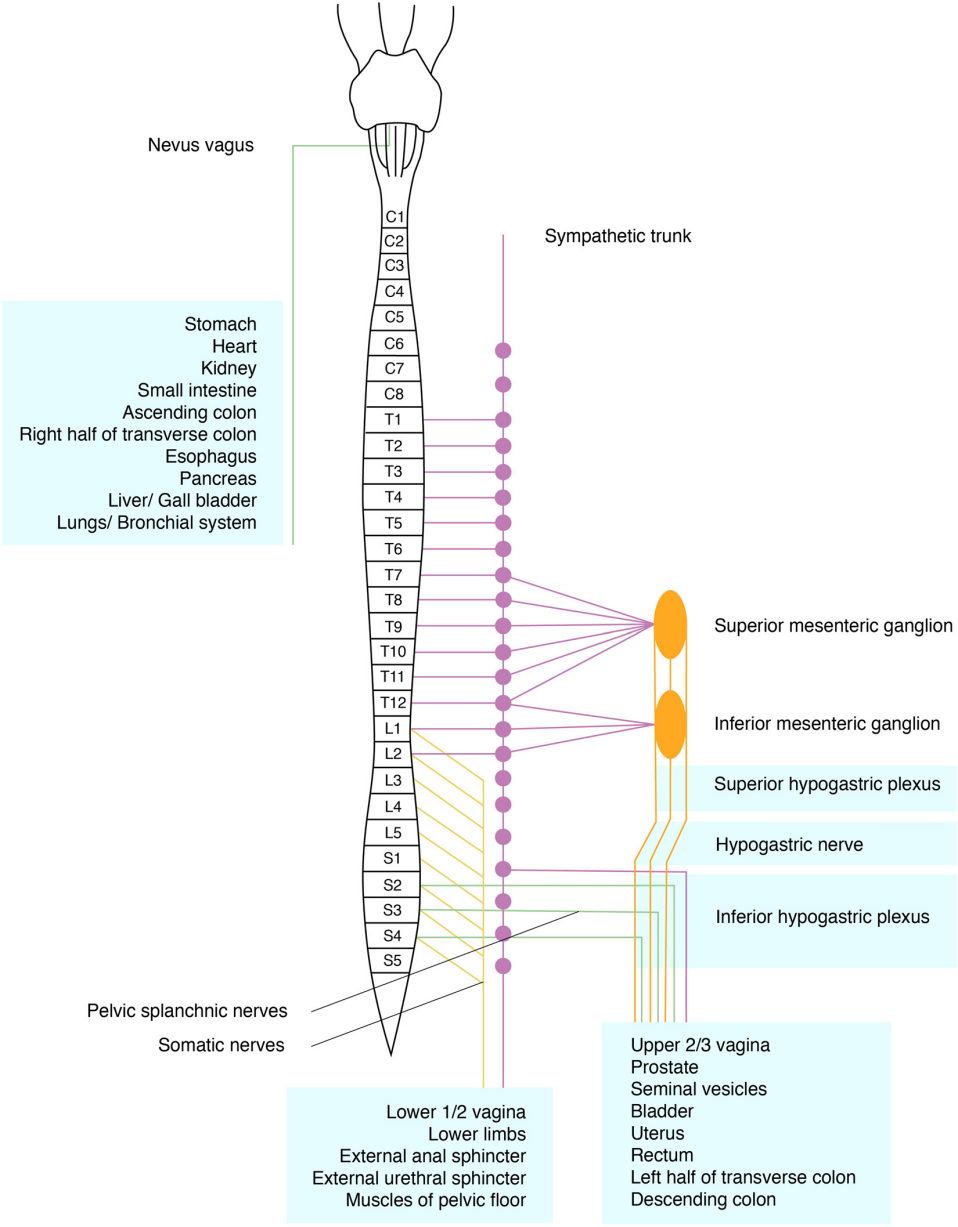
Schematic diagram of somatic and autonomic pelvic nerves.

Pelvic nerves relay impulses to the brain via the spinal cord. These impulses are modulated at various levels in the central and peripheral nervous systems. As elsewhere in the body, the somatic and autonomic nervous systems are involved here. The autonomic (involuntary or vegetative) system is composed of the sympathetic and parasympathetic systems [30] (Figure 2).

Somatic nerves (yellow) arise from the lumbar and sacral spinal cord and supply the pelvic floor muscles, including the external urethral and anal sphincter, the lower abdominal wall, and limbs. The sympathetic system comprises the sympathetic trunk (purple) and the preaortic plexus (orange). The lumbar and sacral sympathetic trunk provides innervation for the pelvis and the lower limbs. The inferior mesenteric ganglion/plexus, superior hypogastric plexus (SHP), hypogastric nerves (HN), and inferior hypogastric plexus (IHP) are the main sources of sympathetic supply to the left colon, anorectum, uterus/vagina, prostate/seminal vesicles, and bladder. The pelvic splanchnic nerves (PSN) (green), which constitute the sacral part of the parasympathetic system, arise from the sacral nerve roots and supply the left colon, anorectum, uterus/vagina, prostate/seminal vesicles, and bladder. The vagus nerve is the source of parasympathetic supply to the remaining visceral organs and gonads [17,30,31,32,33,34,35,36,37,38].

## 3 Autonomic nervous system

### 3.1 Sympathetic nervous system

This part of the autonomic nervous system is activated during stress and controls “fight or flight” responses [30]. With reference to pelvic functions, it prevents micturition and defecation, closes the uterine cervix, and prevents the flow of menstrual blood. The nerve structures that relay the sympathetic impulses to and from the pelvis include the IHP, the HN, the SHP. Impulses move upward from the solar plexus to the brain via the spinal cord (Th10-L3). The sympathetic trunk relays its impulses to the thoracolumbar region of the spinal cord [31].

#### 3.1.1 Sympathetic trunk

The bilateral ganglionated sympathetic trunk extends paravertebrally and is located behind the inferior vena cava on the right side, lateral to the aorta on the left side [32,36]. It receives presynaptic (preganglionic) neurons from the thoracolumbar region of the spinal cord, which synapses within the ganglia of the sympathetic trunk. From this site, postsynaptic (postganglionic) axons travel to the effector organs [31] (Preganglionic and Postganglionic are old terms). Lumbar and sacral postsynaptic sympathetic fibers supply the pelvis and lower limbs [39]. The lumbar sympathetic trunk continues as the sacral sympathetic trunk after entering the pelvis at the sacral promontory on both sides. The sympathetic trunk was found on the ala of the sacrum in just two of 60 cases [32]. The first sacral ganglia are the largest, and subsequently, become smaller or fused in some cases [40]. Postsynaptic sympathetic fibers leaving the sacral ganglia course along with somatic nerves to supply the blood vessels and sweat glands of the lower limbs [30]. The number of nerve fibers joining the corresponding sacral spinal nerve may vary (1–11) [37,41]. Sympathetic nerves in the lower limbs cause vasoconstriction and reduce perspiration on stimulation. Moreover, sacral sympathetic ganglia may give off direct postsynaptic fibers to the pelvic viscera [38].

#### 3.1.2 Sympathetic plexus

Some presynaptic sympathetic fibers from the thoracolumbar region of the spinal cord synapse in ganglia that lie in the prevertebral plexus surrounding the unpaired large vessels of the abdominal aorta. These ganglia sometimes receive presynaptic fibers from the spinal cord via the sympathetic trunk. The names of the individual plexuses within the prevertebral plexus are derived from the underlying anatomical entities. The prevertebral plexus anterior to the aorta is known as the preaortic plexus, consisting of the inferior mesenteric ganglion and the SHP. Postsynaptic fibers travel from here to the organs such as the descending colon, rectum, bladder, and uterus [31].

Sympathetic impulses from the organs may travel directly to the sympathetic trunk, or to ganglia in the prevertebral plexus and then to the sympathetic trunk, or directly to the thoracolumbar region of the spinal cord via the prevertebral plexus. The exact pathways may be difficult to identify for the visceral afferents to the thoracolumbar spinal cord [42].

#### 3.1.3 SHP

The SHP comprises a nervous network extending anterior to the aorta and the sacral promontory and provides postsynaptic sympathetic nerves to the pelvic viscera. The SHP is a continuation of the preaortic and inferior mesenteric plexus [43,44]; it receives branches from the sympathetic ganglia L1–L4, occasionally also from L5 [39,43]. The gross morphology of the SHP varies from a wide reticular meshwork to a single nerve fiber bundle or two distinct nerve trunks running in a parallel fashion along the two sides of the aorta [35,45]. The SHP divides into the left and right HN [17]. However, the level of division is controversial. While most authors have observed the bifurcation at the level of the sacral promontory [36,45,46], a recent study based on 3D magnetic resonance imaging revealed the origin of the HN at the level of the second sacral vertebra [47].

#### 3.1.4 Hypogastric nerve and IHP (Pelvic Plexus)

**Figure 3:**
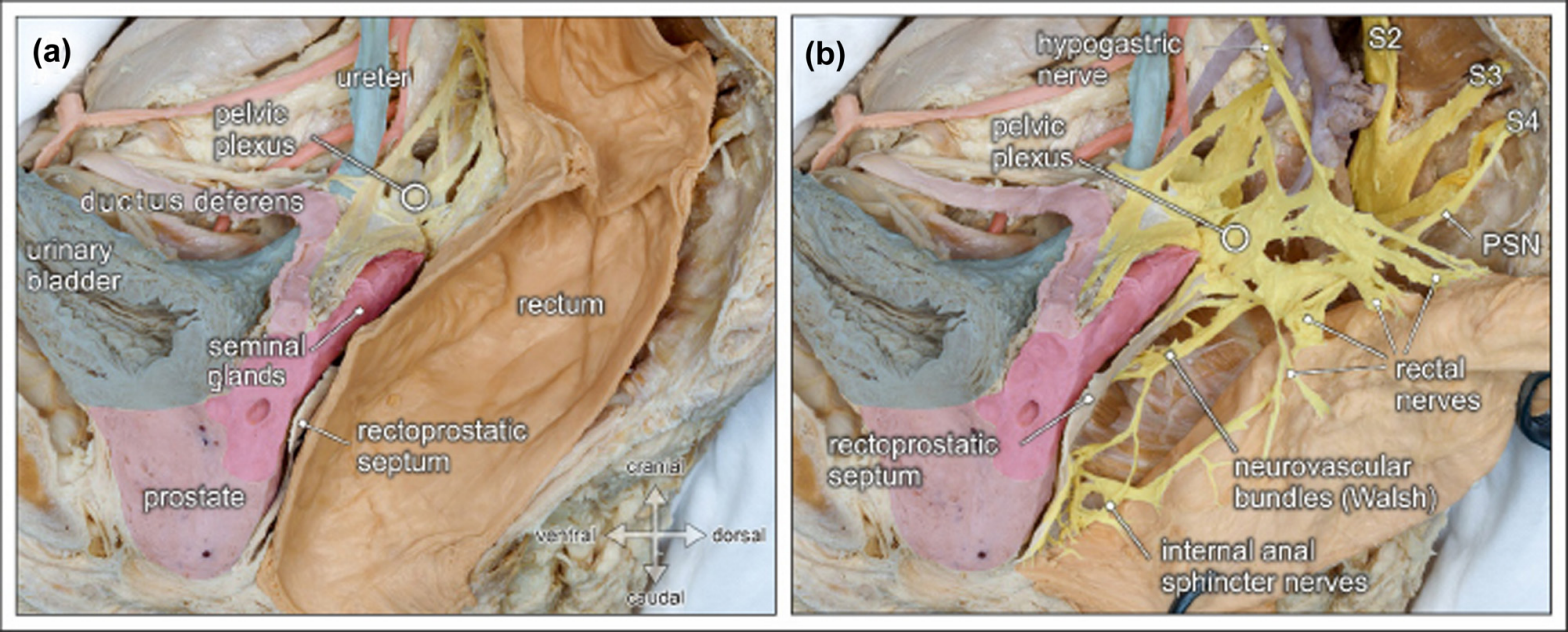
Topographic anatomy of the posterior pelvic compartment. Medial view of a right-sided male hemipelvis. The parietal pelvic fascia is removed to visualize the embedded autonomic pelvic nerves. (a) The posterior pelvic compartment is delimited from the urogenital compartment by the rectoprostatic septum (Denonvilliers’ fascia). (b) The rectum is pulled aside to reveal the inferior hypogastric/pelvic plexus, the hypogastric nerve, and the PSN (PSN, from sacral nerve S4). The inferior hypogastric nerve gives rise to rectal nerves and more caudally to internal anal sphincter nerves, as well as the neurovascular bundle (of Walsh) to the prostate [52].

The HN diverges from the SHP and extends in a caudolateral direction to reach the IHP [36]. The HN runs within the bilaminar parietal pelvic fascia [48] and receives postsynaptic fibers from the sympathetic ganglia (L3–L4), occasionally L1 and L2, the preaortic plexus, and the inferior mesenteric ganglia [39,43]. On their way to the pelvic sidewall, the HN runs approximately 5–20 mm dorsomedially to the ureter, extends posterolaterally to the rectum, and finally, reaches the IHP at the point where the ureter approaches and enters the bladder [17,49] (Figure 3). The HN has primarily sympathetic fibers, but on immunohistochemical stains, cholinergic fibers are also seen, which could arise from the vagus nerve, PSN, or thoracic and lumbar splanchnic nerves [14].

The IHP primarily receives sympathetic fibers from the inferior mesenteric ganglia via the SHP and HN but may also be supplied by fibers from sacral sympathetic ganglia [14,43]. Shiozawa et al. found sacral sympathetic nerve fibers (S1) in only two of six cadavers [50], whereas Baader and Herrmann observed sympathetic fibers in 86% (S2) and 14% (S1) ganglia, respectively [51].

### 3.2 Parasympathetic system

The parasympathetic system is the counterpart of the sympathetic system. As the parasympathetic system is usually active in the resting state, it is also known as the “rest and digest” system. The parasympathetic innervation of the neck, thorax, fore-gut and mid-gut viscera, gonads, uterus, and cervix originates from the vagus nerve (cranial nerve X) and the PSN. The PSN are the main source of parasympathetic nerves to the pelvic viscera and the hindgut. Presynaptic fibers arise from the brainstem (vagus nerve) or the sacral spinal cord segments S2–S4 (PSN), synapse in ganglia located close to or embedded in the organs from which postsynaptic fibers originate, and supply the end organs [30].

#### 3.2.1 PSN

PSN constitute the sacral component of the parasympathetic system, supplying the pelvic viscera and hindgut derivatives. These fibers arise from the sacral spinal nerves S2–4, pierce the presacral fascia, and join the IHP 3–4 cm lateral and 2–4 cm caudal to the rectovaginal or rectovesicle pouch (of Douglas) [36,49,53]. According to Baader and Herrmann, the fibers mainly originate from S3 (60%) or S4 (37%), whereas Shiozawa et al. indicate S2 and S3 as the origin [50,51], and Mauroy et al. indicate S2 (40%), S3 and S4 (60%) but never S1 [46].

#### 3.2.2 Pelvic fascial system and autonomic nerves

The pelvic fascia consists of the parietal and visceral pelvic fascia. Both, sympathetic (SHP, HN, and IHP) and parasympathetic (PSN) nerves are embedded within the dual-lamellar parietal pelvic fasciae, also known as the (pre-)hypogastric sheath or fascia [48,54,55,56]. The IHP extends sagittally at the level of S4 and S5 and is located lateral to the pelvic organs but medial to the pelvic blood vessels [49,51,53]. The rectum lies anterior to the parietal pelvic fascia and is surrounded by perirectal/mesorectal fatty lymphovascular tissue wrapped in the mesorectal fascia. The retrorectal space, extending between the mesorectal fascia and the parietal pelvic fascia, corresponds to the nerve-sparing dissection plane during TME (the “holy plane” of TME, which is a safe plane for dissection to avoid nerve damage) [57] (Figure 3).

**Figure 4:**
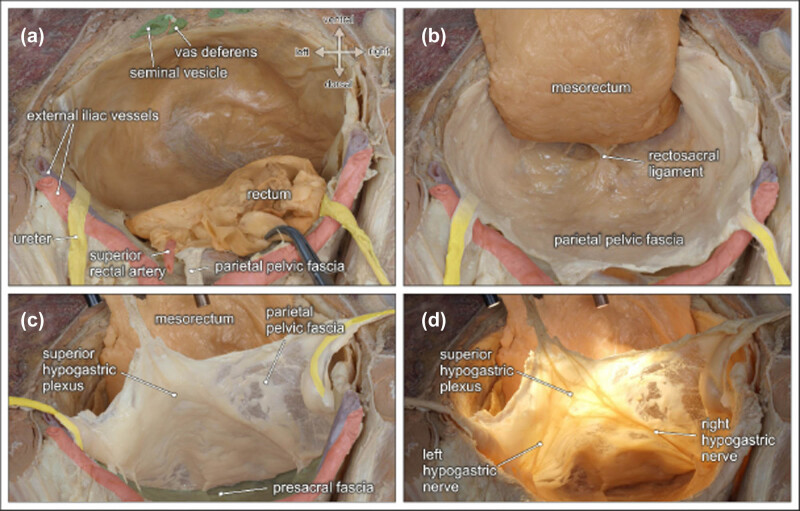
Topographic anatomy of the posterior pelvic compartment. Dorsocranial view of a male pelvis. (a) The rectum and the mesorectum with the superior rectal artery are transected at the rectosigmoid junction (clamp). (b) Dorsolateral mobilization of the mesorectum between the parietal pelvic fascia and the mesorectal fascia along the retrorectal space. Both fasciae are fused by the rectosacral ligament. (c) The parietal pelvic fascia and both ureters are lifted to expose the presacral space behind the presacral fascia. (d) Diaphanoscopy of the parietal pelvic fascia reveals the embedded SHP and both HN [52].

The sacral concavity is covered by the presacral fascia. The presacral fascia that is pierced by the PSN laterally above the levator ani muscle then reaches the IHP [48,54]. The presacral fascia thickens dorsocaudally to the rectum, fuses with the mesorectal fascia, and attaches the rectum to the sacrum, thus forming the rectosacral ligament, which is also known as Waldeyer’s fascia [33,48]. The parietal pelvic fascia extends along the pelvic sidewall and is connected to the pelvic viscera by the parametrium and paracolpium and paracystium [58]. Branches of nerve fibers in this region eventually reach the corresponding organs, namely the rectum, uterus/vagina, bladder, and prostate/seminal vesicles (Figure 4).

### 3.3 Topographic and functional anatomy of the IHP

The IHP is an abundant nerve meshwork. Its main bulk is located adjacent to the vaginal fornix below the broad ligament in females with the cranial part of the IHP located just below the lateral margin of the recto-uterine pouch. In males, it is located close to the lateral rectal wall, in the fibro-connective tissue between the rectum medially and lateral pelvic wall laterally, with the anterior third of the plexus in the rectovesical pouch [14]. Within each pelvic compartment, bundles of nerve fibers diverge from the IHP to form organ-specific plexuses for pelvic autonomic nerves, namely the rectal, uterovaginal, vesicourethral, prostatic, and cavernous (clitoris, penis) plexuses. The posterior part of the IHP supplies the rectum, the anterosuperior part provides nerve fibers for the bladder, and the anteroinferior part supplies the internal anal sphincter and the upper anal canal. According to Mauroy et al. [46], the female IHP is located at the intersection of the uterine artery and ureter; nerve fibers below and close to the ureter travel to the urinary bladder while those alongside and inferior to the uterine artery reach the uterus and vagina. Some vaginal nerve fibers may also pierce the rectovaginal septum. The central portion forms the uterovaginal plexus, extends medially to the uterine artery, laterally to the sacrouterine ligaments, and gives off fibers from its most caudal parts to the vulva and clitoris. In males, the bladder neck fibers from the IHP, lie at 5 and 7 o’clock position at the posterolateral surface. The prostate and cavernous plexus lie between the rectum posteriorly, prostate anteriorly, and levator ani muscle laterally. It has two groups, posterior, between the recta fascia posteriorly and seminal glands anteriorly, and lateral, lateral to the prostate and seminal glands. The posterior group is connected closely with seminal glands and gives fibers to ductus deferens and ejaculatory duct. The lateral group gives fibers to the prostate reaching its apex and external urethral sphincter [14,17,34,44,46,51,53,59].

The pararectal space is an important landmark for the visualization of the IHP [3,7]. The roof of the pararectal space is formed by the posterior leaf of the broad ligament, the floor by the levator ani muscle, the medial boundary by the rectum, and the lateral boundary is formed by the internal iliac vessels [6]. The pararectal space is created by dissecting the lateral parametrium surrounding the rectum and middle of the vagina. The lateral parametrium is divided by the ureter into a lateral parametrium above the ureter, which has the uterine artery and vein, and a lateral parametrium below the ureter, containing the vaginal artery and vein (sometimes called the deep uterine vein). The dorsal paramterium consists of the uterosacral ligament and the dorsal paracolpium consists of the sacrovaginal ligament, sometimes also called the deep uterosacral ligaments. The IHP is embedded in the dorsal parametrium and dorsal paracolpium. Dissection of the pararectal space after lateralizing the ureter provides access to the dorsal parametrium and dorsal paracolpium where the IHP can be visualized. The ventral paracolpium is the deep layer of the vesicouterine ligament that contains the middle and deep vesical veins anastomosing with vaginal veins. This forms the vesical venous plexus closely related to the bladder branches of the IHP [6,58,60].

The functional neuro-topographical anatomy of the IHP was provided by Possover [36]. The upper third of the IHP supplies the vagina, cervix, and uterus, receiving sympathetic fibers from the SHP. Injury to these nervous structures will cause vaginal and cervical hypoesthesia and loss of vaginal lubrication during sexual activity. The middle part of the IHP carries proprioceptive fibers to the bladder and sometimes to the rectum, and is mainly supplied by the sacral sympathetic trunk. The lower third of the IHP is joined by PSN and forms a ventrolateral trunk for innervation of the bladder, and a dorsomedial trunk for motor nerve supply to the rectum [36]. Pain pathways are also likely to follow the nerve routes outlined above. However, an exact topographical mapping of pain-mediating trajectories is yet to be provided. PET studies have yielded evidence of vagal fibers transmitting pelvic visceral sensations directly to the brainstem nuclei [61].

## 4 Somatic nervous system

Ventral branches of lumbar and sacral spinal nerves form the lumbosacral plexus, which ensures somatic nerve supply to the pelvis and the lower limbs. The lumbar plexus comprises the following nerves: the ilioinguinal (L1, L2) and iliohypogastric nerves (T12, L1), the genitofemoral nerve (L1, L2) including its genital and femoral branches, the lateral femoral cutaneous nerve (L2, L3), the femoral nerve (L2, L3, L4), and the obturator nerve (L2, L3, L4). The L4 and L5 roots of the lumbar plexus join the first sacral nerve root (S1) to form the lumbosacral trunk. The lumbosacral trunk/sacral plexus is the source of the pudendal nerve (S2, S3, S4), the superior (L4, L5, S1) and inferior gluteal nerves (L5, S1, S2), the sciatic nerve (L4, L5, S1, S2, S3), and the posterior femoral cutaneous nerve (S1, S2, S3). Timoh et al. conducted a computer-based anatomical dissection study of seven female fetuses, and immunostained the specimens, to demonstrate the nature and function of the levator ani muscle innervation. One of the conclusions was that the pudendal nerve was both somatic and autonomic, located below the levator ani muscle, as opposed to the general thought that the pudendal nerve is purely somatic [16]. All somatic nerves are accompanied by postsynaptic sympathetic fibers from the lumbar and sacral sympathetic chain to supply the smooth muscles of the blood vessels, sweat glands and apocrine glands, andarrector pili muscle of hair follicle of the skin, and also the bone marrow (which modulates the immune system) of the corresponding regions [34,62,63,64].

**Figure 5:**
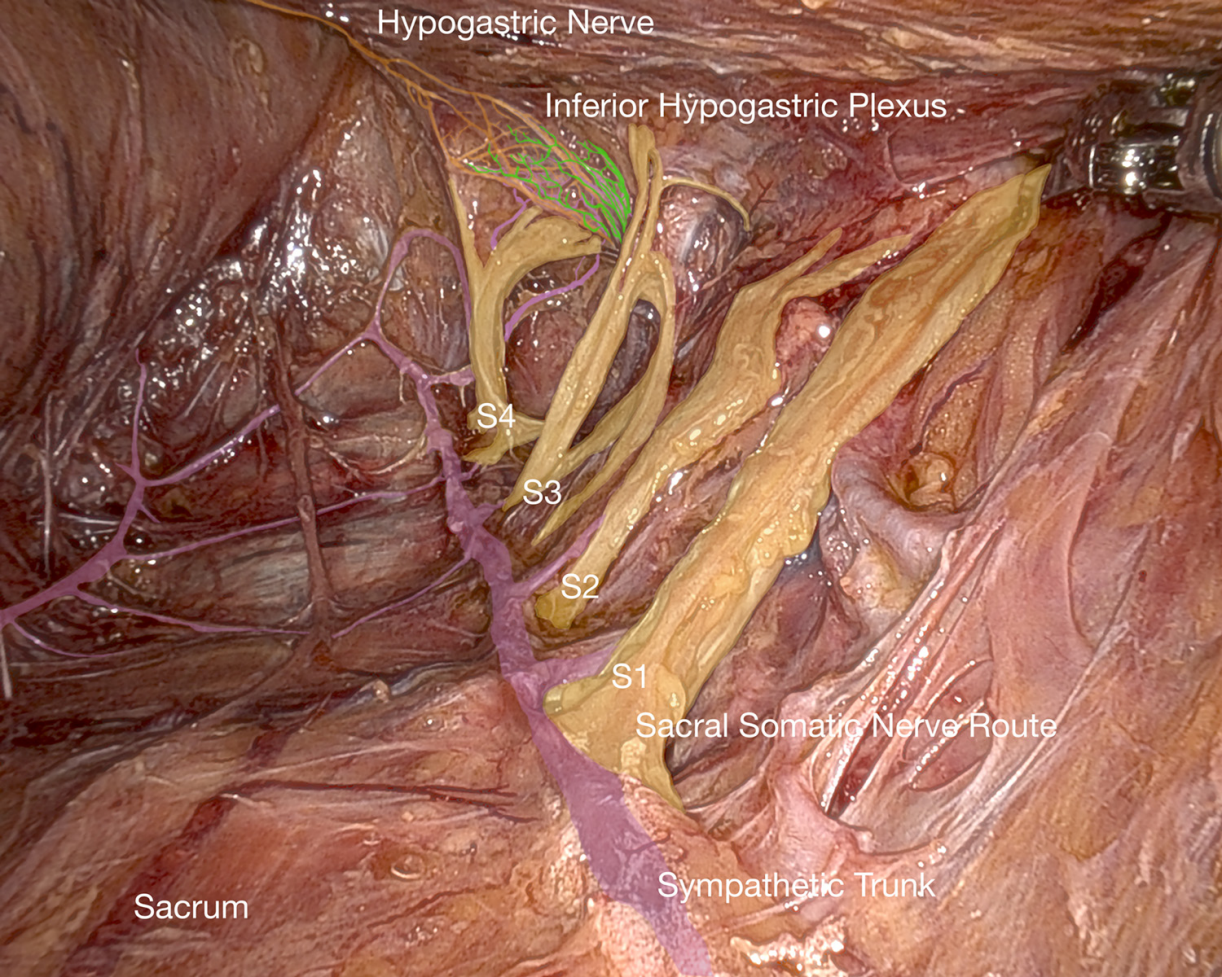
Robotic surgery performed in a female body donor, exposing the sacral nerve roots (yellow) S1–S4, the sacral sympathetic trunk (purple), the hypogastric nerve (orange), and PSN (green).

**Figure 6:**
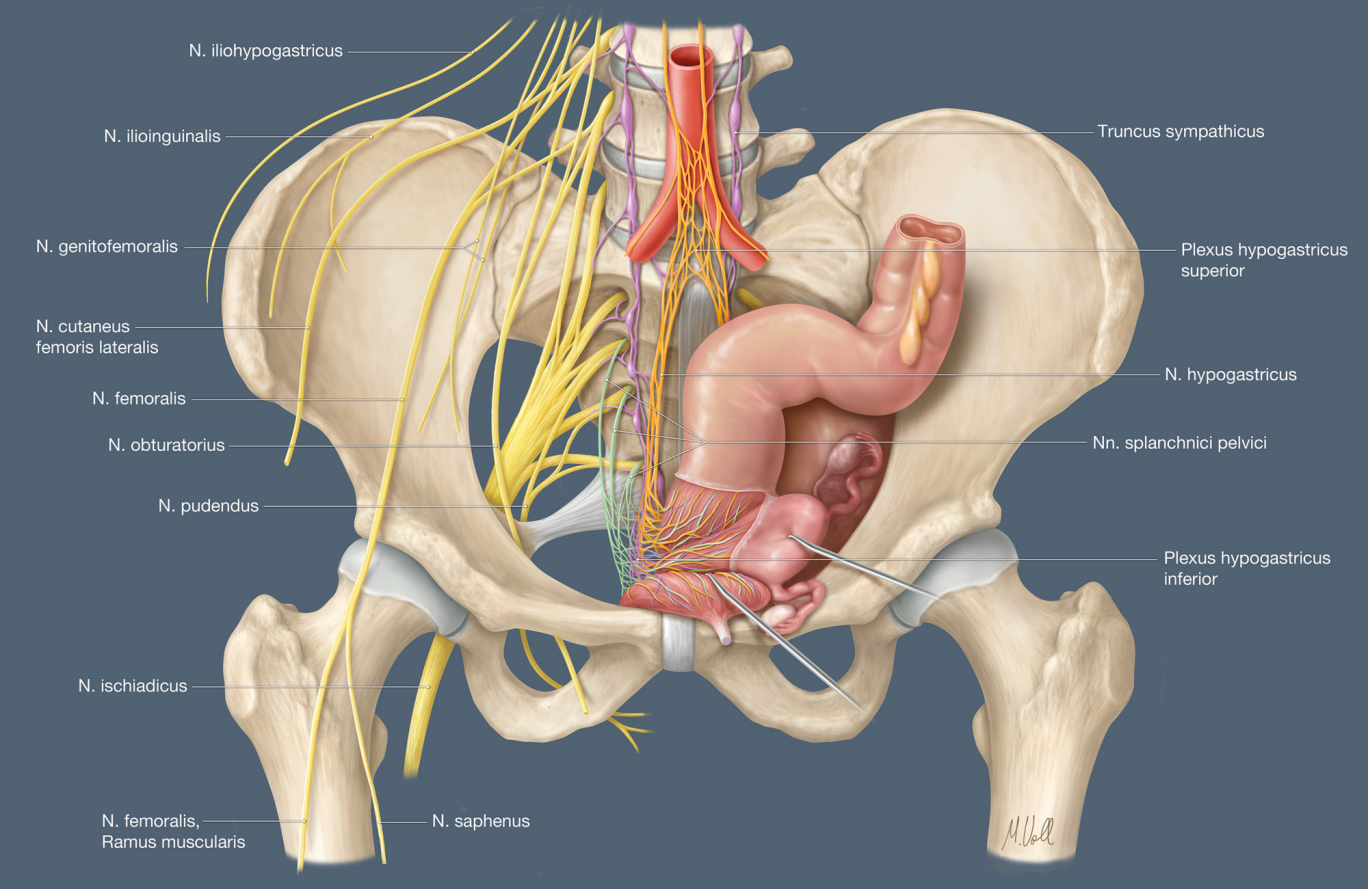
Anatomical illustration of somatic and autonomic pelvic nerves in a female pelvis (frontal view). Yellow – somatic nerves, orange – sympathetic plexus, purple – sympathetic trunk, green – PSN.

The sacral nerve roots leave the sacrum via the ventral sacral foramina to form the sacral plexus and its peripheral nerves on both sides in front of the piriformis muscle. To expose this region in front of the sacral concavity, we performed robotic surgery using the da Vinci Xi (Intuitive Surgical Inc., Sunnyvale, CA) in a body donor at the Kurt Semm Academy for laparoscopy and robotic-assisted surgeries, UKSH, Kiel, Germany. After detachment of the presacral fascia and identification of presacral blood vessels, we were able to demonstrate the sacral nerve roots S1–S4, the sacral sympathetic trunk, and the PSN (Figure 5). Figure 6 provides an overview of somatic and autonomic pelvic nerves (Figure 6).

## 5 Functional involvement of pelvic nerves in defecation, micturition, sexual activity, and pelvic pain

Pelvic nerves are closely related to the mechanisms of defecation and micturition, as well as chronic pelvic and visceral pain. They are also responsible for complex sexual activity involving genital arousal, orgasm, and ejaculation [65]. Sympathetic pelvic nerves are involved in the mediation of proprioceptive sensation [34,66] as well as pain [36] from the upper pelvis via the HN and the SHP to the sympathetic trunk [20]. Efferent sympathetic nerve fibers evoke contraction of the internal urethral and anal sphincters and exert relaxation on the visceral nonsphincter smooth muscle. Parasympathetic pelvic nerves also carry nociceptive sensations to the sacral dorsal root ganglia from the pelvic viscera, such as the vagina, cervix, and bladder, and mediate contraction of visceral smooth muscles [20,34,67].

### 5.1 Defecation and micturition

The acts of defecation and micturition are a complex interplay of both, central and peripheral nerves. Cortical control of micturition and defecation is specific to humans. The neural pathways involved in defecation and micturition are described in Table 1.

**Table 1:** Defecation and micturition pathways [68,69,70]

	Afferent pathway	Efferent pathway
First-order neuron	Visceral afferent nerves from the bladder, rectum, and colon traverse via sympathetic/parasympathetic nerves to the dorsal root ganglion and the dorsal horn of the spinal cord, and then to the intermediolateral cell column of the sacral spinal cord	Higher centers like the thalamus, hypothalamus, and cortex send impulses to Barrington’s nucleus (BN) and the lateral cell group (LCG), depending on social and emotional factors
Second-order neurons	Several interneurons in the spinal cord synapse in the spinal cord to send impulses to Barrington’s nucleus (BN) in the brainstem	Impulses from the BN (pontine defecation and micturition center) are sent to the intermediolateral cell column of the sacral spinal cord, which controls the autonomic pathways and smooth muscles of the colon, rectum, and bladder
		The LCG sends impulses to Onuf’s nucleus in the sacral spinal cord, which controls motor functions of the external anal and urethral sphincter
Third- and fourth-order neurons	From the BN to the thalamus, PAG, cingulated gyrus, and medial frontal cortex	Sympathetic nerves relax the rectum and bladder via the HN and plexus, and contract the internal anal sphincter and internal urethral sphincter. Parasympathetic nerves contract the colon, rectum, and bladder via the PSN. The external anal and urethral sphincter is contracted by the pudendal nerve (somatic)

The pontine defecation and micturition center is located in BN in the brainstem, although the neurons that control the bowel and bladder have not been differentiated yet on the basis of their size, morphology, position, and chemistry [69]. The periaqueductal gray area (PAG) has multiple connections with the thalamus, hypothalamus, insula, anterior cingulated gyrus, and cortex. These higher centers are responsible for the voluntary decision of defecation or micturition in accordance with social appropriateness [71].

### 5.2 Sensory pathways from the pelvis

Sensations from the pelvic viscera travel via autonomic pelvic nerves, whereas those from somatic regions such as the perineal, pubic, or gluteal regions travel via somatic nerves [72]. Somatic afferents other than pain and temperature have their first-order neuronal cell body in dorsal root ganglion. “(Note, there are no synapses in a dorsal root ganglion, unless aberrant synapses may be present in chronic pain where presynaptic sympathetic nerves may synapse with nociceptive pseudounipolar cell bodies [73]).” The first-order axon enters the dorsal horn, travels up the fasciculus gracilis, and synapses on the second-order neuron in the nucleus gracilis in the caudal medulla. Second-order axons decussate to enter the medial lemniscus that ends in the ventral posterolateral (VPL) nucleus of the thalamus. Here, it synapses on the third-order neuron. The third-order axon travels through the posterior limb internal capsule to enter the primary sensory cortex where it will synapse at the medial aspect of the postcentral gyrus called the paracentral lobule [74].

Pain and temperature sensations have different pathways. Fast pain travels via the neospinothalamic pathway, whereas slow pain travels via the spinoreticulothalamic pathway. The first-order neuron for fast pain is in the dorsal root ganglion. The first-order axon enters the dorsal horn, collateralizes up and down several spinal cord segments, synapsing on the second-order neuron in the dorsal horn, mainly lamina I and probably substantia gelatinosa. Second-order axon (spinothalamic) decussates to form the spinothalamic tract/anterolateral system that ends in the VPL nucleus of the thalamus. The third-order axon travels through the posterior internal capsule to reach the paracentral lobule [74].

Slow pain is a multisynaptic pathway. The first-order neuronal cell body is in the dorsal root ganglion, the first-order axon enters the spinal cord, collateralizes up and down several spinal cord segments, and synapses in laminae IV and V, VII and X on presynaptic sympathetic and parasympathetic neurons [75,76]. The second-order axon decussates in the spinal cord, travels to the reticular formation and PAG via the multisynaptic pathway, eventually ending in the medial aspects of the thalamus and synapsing with third-order neurons, which then activates the entire nervous system. The third-order axons eventually project to the cerebral cortex [74].

The stimuli are processed in the brain, perceived as nociceptive, proprioceptive, or touch sensations. They are projected downward to the viscera via the spinal cord, sympathetic trunk and plexus, PSN, and somatic nerves. Several regions in the brain, brainstem, and spinal cord are connected via interneurons, which play an important role in modulating these impulses [74,77].

The Gate theory of pain control was proposed to explain the relief of pain by acupuncture, massage, and application of liniments to painful areas. It was suggested that pain fibers were inhibited at the entry point in the central nervous system by fibers carrying nonpainful stimuli like touch and pressure, thus closed the gate for pain [78]. The analgesia system suggests that areas in the brainstem like PAG, periventricular area of the diencephalon can block pain sensations via the reticulospinal tract to the posterior gray column of the spinal cord. The release of endorphins and enkephalins in the posterior gray columns could serve to inhibit analgesic substances like substance P, thus regulating the nociceptive input [74,79].

### 5.3 CPP

The prevalence of CPP in women is considerably high (5.7–26.6%) [80] and men who are examined for chronic prostatitis, 90% are diagnosed to have CPP [18]. Pathophysiology for CPP has been extensively investigated, although no single theory has been able to explain the syndrome. Much has been attributed to visceral pain. Etiology is multifactorial, both in men and women (Table 2). Many central and peripheral mechanisms are involved, thus proving that “pain is not only in the head” [81]. Chronic changes occur in the body and make pain a permanent phenomenon.

**Table 2:** Etiology for CPP [82,83]

Urological	Interstitial cystitis, chronic sphincter dyssynergia
	Bladder cancer and therapy, chronic or complicated urinary-tract infection, painful bladder syndrome, urethral diverticulum
	Men: prostatitis, epididymitis, orchitis
Gynecological	Adenomyosis, endometriosis, leiomyoma, adnexal mass, ovarian remnant syndrome, pelvic adhesions, vestibulitis, vulvodynia
Gastrointestinal	Inflammatory bowel disease, irritable bowel disease, colonic diverticulitis, colorectal cancer
Neurological	Nerve entrapment syndromes, injuries secondary to surgeries,
	neural tumors, compression of nerves by adjacent tumors, spinal cord injuries
Orthopedic	disc prolapse, spinal canal stenosis, chronic hip disorders,
	bone malignancies
Somatoform disorders	Complex functional disorders
Psychiatric	Depression, anxiety disorders, abuse – sexual, emotional, physical

The terms used to describe dysfunctional sensations in the pelvis could be a pain, itching, or persistent arousal, which follow the same neurological pathways [84]. Multiple neuroreceptors, neurotransmitters, and inflammatory mediators are involved in the generation of pelvic pain. Several changes, e.g., central sensitization, take place over time and can make the alterations permanent [67]. Receptors in the pelvic viscera are polymodal in character, including mechanoreceptors, thermoreceptors, chemosensitive, and nociceptive receptors. Although these receptors mediate different types of stimuli, they are all capable of inducing the perception of pain in the cerebral cortex. Whereas acute pain is usually caused by the activation of high-threshold nerve fibers, chronic pain is provoked by continuous stimulation of low-threshold fibers. In contrast, silent nociceptive fibers are only activated by strong algogenic stimuli [85].

Inflammatory mediators such as calcitonin gene-related peptide (CGRP), nerve growth factor, or substance P are released in the CNS and periphery, and sensitize nociceptors or trigger nearby cells to release other activating substances that stimulate nerve fibers in a feedback loop-like pattern. This continuous stimulation lowers the threshold for excitation, and thus, leads to hypersensitization at the peripheral and central levels. It also induces the growth of new nerve fibers and receptors that maintain the loop and contribute to CPP [19,22,86]. Chronic inflammation causes catecholamine overactivity, leading to muscular spasms and pain. Cytokines released from the pelvis can cross the blood– brain barrier, adding to mental health problems and pain memory [18]. Pain catastrophizing may occur resulting in a coping response to pain that amplifies the magnitude of pain [87]. Long-term pain causes expansion of the somatosensory cortex representing the pain, and with time, the nonpainful stimulus will lead to pain. Chronic pain will leave memory traces in the brain. This alters the behavioral pattern of the individual via cortical reorganization, which expands the pain processing area of the cortex. There is sprouting of new axons and neuroplasticity that develop pain memory [23].

Somatic and visceral nociception mediated by unmyelinated or lightly myelinated fibers often converges at the same spinal level. This viscerosomatic and viscerovisceral convergence could explain the diffuse nature of visceral pain, muscular spasms, and overlapping symptoms of CPP. Painful sensations carried from the viscera might stimulate somatic afferents and efferents, causing pain in the corresponding dermatome and muscle contraction in the corresponding myotome. Pain in the visceral organ may evoke referred pain in the lower limbs via sacral nerves, or in the lower back, pubic and inguinal region via lumbar nerves. Gonadal pain usually causes referred pain in the T10–T11 dermatomes [19,20].

Despite an abundance of sensory fibers from the skin, cutaneous nerve fibers are less numerous than visceral nerve fibers in the intramedullary aspect. Second- and third-order neurons responsible for visceral afferents exhibit more extensive arborization than somatic afferents in the spinal cord, and terminate at several spinal levels. This is responsible for the diffuse nature of visceral pain and subsequent referred pain. A single visceral primary afferent fiber may innervate two organs; two afferent fibers from the different pelvic organs may converge on the same dorsal root ganglion, causing organ crosstalk and several organ symptoms [19,20,21,22,85].

Since the motor and sensory pathways from pelvic organs converge at the spinal level, neurotransmitters can be released onto the visceral targets through both pathways. Hence, multiple sensory and motor symptoms may exist simultaneously in patients with pain and complaints in the bowel or bladder.

### 5.4 Sexual activity

The act of sexual intercourse is complex. Although not completely understood, various central and peripheral interactions play a role in the whole act. It involves physiological, psychological, social, and emotional factors [88]. Neurologic control of sexual responses is by all three pelvic nerve systems, with descending excitatory and inhibitory control by the central nervous system. Multiple areas in the brain, cerebrum, and midbrain are involved. The afferent sensory pathway involves the hypogastric, pudendal, and vagus nerve, which relays in the spinal cord and midbrain [89]. The efferent motor pathway involves the sacral parasympathetic and the pudendal nerve [65]. Sexual behavior is also modulated by hormones under gonadal control [89].

Genital arousal is a dynamic process, which can have three components, psychogenic generated from thoughts, reflexogenic generated from genital stimulation, and nocturnal (rapid eye movement or REM sleep). The endpoint is penile erection or clitoral tumescence. This involves parasympathetic activity causing secretions of vasodilatory neurotransmitters like nitric oxide (NO), CGRP, vasoactive intestinal peptide, and prostaglandins with the relaxation of smooth muscle to override the sympathetic activity of detumescence [65,90]. The final common pathway through which this is achieved is the pelvic nerves, which receive input from the medial preoptic area and genitals (activation of the pudendal nerve), and get modulated at the spinal center of T11–L2 (psychogenic) and S2–S4 (reflexogenic) [91,92]. It is mainly the parasympathetic component involved in tumescence but the sympathetic nervous system also has some proerectile function [65]. Nocturnal erections may occur due to the activation of cholinergic neurons with inhibition of serotonergic neurons during REM sleep [93].

Parasympathetic nerves also stimulate the bulbourethral glands to secrete clear viscous fluid, which lubricates the penis and increases the urethral pH. This is the secretion phase. Sympathetic nerves contract smooth muscles of the epididymis, ductus deferens, seminal glands, and prostate to release secretions (semen) into the bulbar urethra. This is the emission phase. Simultaneously, the internal urethral sphincter contracts under sympathetic control. This prevents retrograde ejaculation. Somatic nerves stimulate the pelvic floor muscles (ischiocavernosus, bulbospongiosus, superficial and deep transverse perineal muscles, external anal sphincter, and the pelvic diaphragm) to maintain rigidity. Rhythmic contractions of the bulbospongiosus cause semen to release into the spongy urethra causing ejaculation. After ejaculation, the sympathetic overactivity overrides the parasympathetic activity to maintain detumescence again [92].

Orgasm, which is the release of neuromuscular tension and pelvic vasocongestion, involves complex neural circuits that are not completely understood. It is usually perceived as a pleasurable activity, depends on the time interval from the last sexual activity, degree of excitement, and psychosexual make of the mind. Orgasm usually experienced with ejaculation can be experienced even without it, and thus they involve different neurophysiologic circuits. Both sympathetic and parasympathetic systems at peripheral and central levels are involved [65,92,94].

## 6 Conclusion

Pelvic nerves are complex. Their anatomy and physiology must be comprehended as a complete unit in order to understand the pathophysiology of pelvic diseases. Several mechanisms have been proposed to explain visceral pain, yet do not fully explain the complex neural mechanisms of this condition. The usual understanding of HN as sympathetic and PSN as parasympathetic is not entirely correct due to mixed components found in both. Likewise, the pudendal nerve being purely somatic is also not true. Its physiological and pathological implications are still unclear. Any treatment of CPP must be based on a comprehensive understanding of anatomy and physiology. The actual causes and cure of pelvic pain still elude our knowledge. Theories underlying ancient treatments such as acupuncture or yoga might provide insights into the treatment of neurological disorders.

## Appendix A

History and examination sheet for a neuropelveological diagnosis.

**History**

Pain

**Table:**

**Somatic**	**Visceral**
Specific, burning pain	Vague, dull, hypersensitive bladder and colon
Can often be pinpointed	Cannot be pinpointed
Associated with pelvic motor dysfunction and irradiation to the dermatome	Associated with vegetative symptoms: anxiety, pallor, nausea, vomiting, fatigue, syncope, dry mouth, tachycardia, irritable
**Neurogenic**	**Non-Neurogenic**
Axonal damage	No-axonal damage
Hypo symptoms – e.g., hypoaesthesia, chronic constipation	Hyper symptoms – e.g., hyperaesthesia, irritable colon

- Aggravating factors: sitting/standing/walking.
- Previous history of surgeries: (a) which surgery, (b) symptoms before/after surgery; time since surgery symptoms started.
- Bladder complaints: polyuria/nocturia/urge/stress incontinence/difficulty in start of micturition/dysuria/burning micturition/retention of urine.
- Bowel complaints: chronic constipation/irritable bowel/painful defecation/fullness of abdomen/tenesmus/alternate constipation/diarrhoea.
- Lower limb symptoms: pain at a specific point or area/while moving, motor dysfunction.
- Sexual symptoms: increased or decreased libido/dyspareunia-deep or superficial/persistent arousal.
- Any of the symptoms are cyclical?.
- Psychosocial factors: sexual abuse/depression/childhood trauma/drug abuse.

**Examination**

- Dermatomal testing: to elucidate area of affect, will tell which nerve root is affected.
- Motor movement testing of each muscle: elucidates which nerve root is affected.
- Palpation of some superficial nerves like the pudendal nerve, the sciatic nerve.
- Sonography: probe pain, doppler to detect lesions.
- Urodynamic testing: for bladder hypersensitivity/overactive bladder/urge incontinence.
- Pelvic neuro MRI: to see any specific nerve growths.
